# No evidence that nest site choice in Pied Flycatchers is mediated by assessing the clutch size of a heterospecific, the Great Tit

**DOI:** 10.1007/s10336-021-01900-1

**Published:** 2021-05-29

**Authors:** Tore Slagsvold, Karen L. Wiebe

**Affiliations:** 1 Centre for Ecological and Evolutionary Synthesis (CEES), Department of Biosciences, University of Oslo, Oslo, Norway; 2 Department of Biology, University of Saskatchewan, Saskatoon, Canada

**Keywords:** Egg covering, *Ficedula*, Nest site preferences, *Parus*, Social learning, Tutor

## Abstract

Among species that use similar resources, an individual may benefit by observing and copying the behavioural decision of a heterospecific. We tested the hypothesis of heterospecific social learning in passerine birds, namely that a migrant species, the Pied Flycatcher *Ficedula hypoleuca,* uses external markings on the nest cavities of a resident species, the Great Tit *Parus major*, as cues when choosing a nest site. Others have suggested that prospecting flycatchers assess the clutch size of tit “demonstrators” by entering their nest boxes and, assuming that a large clutch indicates a high-quality individual, will copy the nest appearance of tits with large, but not small clutches. During a 4-year period in Norway, we designed a similar study but did not find that flycatchers based their nest choice on the clutch size of tits. Neither were there any relationships between clutch size of the tit and its laying date, incubation behaviour, or the number of eggs visible through nest material during egg-laying so Pied Flycatchers did not use these indirect cues to assess quality of the tutor. Filming of tit nests showed that prospecting flycatchers did not enter tit nest boxes to assess the content. Indeed, incubating female tits only left their nest boxes for short bouts of unpredictable duration so there was little opportunity for flycatchers to inspect the nest contents unnoticed. Our study calls into question the mechanism of using the content of tit nests as public information for choosing traits of nest sites based on external characteristics. We suggest that similar studies of nest site choice in relation to possible social information transfer be replicated more widely.

## Abstract (de)

**Keine Belege dafür, dass Trauerschnäpper ihren Nistplatz aufgrund einer Einschätzung der Gelegegröße einer anderen Art, der Kohlmeise, auswählen.**

Wo Arten ähnliche Ressourcen nutzen, kann es für ein Individuum vorteilhaft sein, das Entscheidungsverhalten eines Artfremden zu beobachten und zu kopieren. Wir prüften die Hypothese des heterospezifischen sozialen Lernens bei Singvögeln, hier konkret, dass eine Zugvogelart, der Trauerschnäpper *Ficedula hypoleuca*, äußere Kennzeichen an den Nisthöhlen einer Standvogelart, der Kohlmeise *Parus* *major*, als Entscheidungshilfen bei der Nistplatzwahl nutzt. Andere Autoren haben vermutet, dass auskundschaftende Fliegenschnäpper die Gelegegröße der als Vorbild dienenden Meise einschätzen, indem sie in den Nistkasten schlüpfen und in der Annahme, dass ein großes Gelege ein Individuum von hoher Qualität signalisiert, ihr Nestäußeres an dem von Meisen mit großen, nicht jedoch kleinen Gelegen ausrichten. Über einen Zeitraum von vier Jahren entwickelten wir in Norwegen eine ähnliche Studie, konnten aber nicht feststellen, dass die Fliegenschnäpper ihren Nistplatz anhand der Gelegegröße der Meisen auswählten. Ebenso wenig gab es Zusammenhänge zwischen der Gelegegröße der Meise und dem Legedatum, Brutverhalten oder der Anzahl der während der Eiablage durch das Nistmaterial hindurch sichtbaren Eier, so dass die Trauerschnäpper diese indirekten Hinweise auf die Qualität ihres Vorbildes nicht nutzten. Filmaufnahmen von Meisennestern zeigten, dass die auskundschaftenden Fliegenschnäpper nicht in die Nistkästen schlüpften, um den Inhalt zu begutachten. Tatsächlich verließen die brütenden Meisenweibchen ihre Nistkästen nur für kurze Zeiträume unvorhersehbarer Dauer, so dass die Fliegenschnäpper wenig Gelegenheit hatten, den Nestinhalt unbemerkt in Augenschein zu nehmen. Unsere Untersuchung stellt den Mechanismus infrage, dass der Inhalt von Meisennestern als frei zugängliche Information dient, um Nistplatzeigenschaften anhand von äußeren Merkmalen auszuwählen. Wir empfehlen, ähnliche Studien zur Nistplatzwahl im Hinblick auf möglichen sozialen Informationstransfer in größerem Rahmen zu wiederholen.

## Introduction

Animals that use information from other species when making their own breeding decisions face the challenge of trying to assess or predict behaviour, of a species that is quite different from themselves but which uses a resource in a way that is worth copying. Temperate bird species may have limited time to find food, high-quality habitats, mates or safe nest sites, so to increase search efficiency, they may use information gained from observing conspecifics (Slagsvold and Wiebe 2011; Aplin et al. 2015; Whiten et al. 2017), and heterospecifics (Camacho-Cervantes et al. 2015; Webster and Laland 2017; Morinay et al. 2018). The use of heterospecific information may confer an advantage if it results in locating high-quality habitats and common food resources, or in avoiding shared potential dangers, such as predators (Danchin et al. 2004; Parejo et al. 2005; Magrath et al. 2015). However, interacting with other species to obtain information may be costly if it risks injury from a species that is stronger or more aggressive.

Some studies of passerine birds provide strong support for information transfer among animal species in the wild (e.g., Avarguès-Weber et al. 2013; Slagsvold et al. 2013; Farine et al. 2015). This includes a series of experiments showing that migratory species, which often face strong time constraints on breeding, use cues from resident species when deciding where to settle (Forsman et al. 2002, 2008; Thomson et al. 2003). For instance, migrants may use the phenology of resident birds in the choice of local nesting habitat type (Samplonius and Both 2017). Birds, especially late-arriving migrants, may also use cues from resident birds when choosing traits of a nest site (Seppänen et al. 2005; Kivelä et al. 2014; Morinay et al. 2020a).

A version of the Selective Interspecific Information Use hypothesis (SIIU; Forsman et al. 2018) suggests that a migrant species chooses a nest cavity with an external appearance that matches that of a local resident if the resident seems to be successful. The hypothesis assumes that a large number of eggs and nestlings reflects the quality of the choice of the resident and is the cue that is assessed by the migrant when choosing a nest cavity (Seppänen et al. 2011). Thus, the choice of a nest site is thought to be based on associative learning, the migrant choosing a similar-looking nest site when the “demonstrator” has a large but not a small clutch or brood.

This version of the SIIU hypothesis has received much focus within a model system of migrant Pied Flycatchers *Ficedula hypoleuca* and resident Great Tits *Parus major* (Avarguès-Weber et al. 2013; Aplin 2016; Szymkowiak 2019), but we recently pointed out that its acceptance is premature because the mechanism by which one species of bird supposedly assesses the clutch and brood size of another has not been well articulated or studied. We also questioned the adaptive significance of copying the external markings around cavity entrances when choosing nest sites (Slagsvold and Wiebe 2017, 2018). Looking inside the nest cavity of a heterospecific competitor may be costly. If a flycatcher enters a tit box when the female is out on an incubation recess, the flycatcher may be killed when the tit returns (Merilä and Wiggins 1995; Samplonius and Both 2019) so it is not clear whether a strategy of assessing heterospecific clutch and brood size can be expected to evolve.

Here, we tested the hypothesis with a similar experimental design to that used by Seppänen et al. (2011), letting Pied Flycatchers choose between a dyad of empty nest boxes with an exterior painted marking that was similar, or not, to that painted on a nearby nest box of a Great Tit. We had five objectives: We first tested whether nest site choice by Pied Flycatchers is related to Great Tit clutch and brood size in our study area in Norway. We included age and laying date of the female flycatcher in the analysis because inexperienced, late-arriving birds may be more likely to use information from resident birds in their nest site choice (Seppänen and Forsman 2007). We also studied whether more Pied Flycatchers settled at a trial site when Great Tit clutch size was large rather than small. Second, because the symbol choice by flycatchers was not related to tit clutch size (see results), we investigate by video filming whether prospecting flycatchers actually entered tit nests. By filming incubation rhythms of tits, we quantified the time available for prospecting flycatchers to enter tit nests unnoticed by the owner. Third, we studied whether Pied Flycatchers can use indirect cues to assess tit quality. Hereto, we compared attentiveness of the female Great Tit on the nest, male feeding rate of the incubating female, and tit nesting phenology (date of tit egg-laying) with final tit clutch size. Fourth, because we found no relationship between these indirect cues and clutch size (see results), we tested whether final tit clutch size itself can be assessed by prospecting Pied Flycatchers during the egg-laying period when clutch size is incomplete and when the tits spend much time outside the nest cavity and when flycatchers have been observed to inspect their nest (Forsman et al. 2018). This is not obvious because at this stage of nesting, the tits often cover the eggs when leaving the nest (Haftorn and Slagsvold 1995; Loukola et al. 2020a). Fifth, we documented the extent of variation in Great Tit clutch size among females and study years to assess whether many tit nests need to be visited by prospecting Pied Flycatchers to obtain a reliable assessment of the relative quality of a local demonstrator tit in the population.

## Materials and methods

### Study area and study species

The study was done near Oslo, Norway, in managed woodlands with mixed deciduous and coniferous trees provided with nest boxes. Most Great Tits are resident here whereas the Pied Flycatcher is a long-distant migrant arriving in the area from late April through May. Male flycatchers occupy a nest cavity soon after arrival which they display to prospecting females often after many tits have started incubation (Lundberg and Alatalo 1992). Great Tits (~ 17 g) are larger than Pied Flycatchers (~ 12 g) and in both species, only the female builds the nest and incubates the eggs. All the wooden nest boxes were placed about 1.5 m above ground on live trees.

We conducted a total of 96 symbol trials (16 in 2016, 13 in 2017, 28 in 2019 and 39 in 2020). In 2016, the trials were conducted in a study area at Dæli (14 ha; 1.6 km^2^, 59 °56 ′N, 10 °32 ′E) where an excess of nest boxes has been provided at fixed sites since 1995. In 2016, there were 534 nest boxes available and we found 76 first nest attempts of Great Tits and 46 of Pied Flycatchers (at least one egg laid). We selected 16 active tit nests that were at least 80 m from the nearest conspecific and in which the resident pair was either egg-laying or incubating when the flycatchers arrived. The study area was selected to simulate an unmanaged forest with an excess of natural nest cavities (Czeszczewik and Walankiewicz 1999). The rest of the symbol trials were conducted at “solitary” sites in woodlands where no nest boxes had been available previously and where there were few natural cavities. These boxes to attract Great Tits were erected in mid-March each year. In 2019, we used 10 of the same solitary sites as in 2017 whereas 17 were new; in 2020, we used 21 of the same solitary sites as in 2019 whereas 18 were new.

We took GPS recordings of each trial site to calculate distances between them. At the sites where a Pied Flycatcher built in a nest box of a dyad (*n* = 54), the distance to the next nearest tit nest at the nearest trial site averaged 256 ± 277 m (SD). The trial sites of previous studies were at least 1 km apart (Seppänen et al. 2011). The shorter distances between our sites were useful because we wanted to know whether prospecting Pied Flycatchers actually enter tit nest cavities to assess clutch size and greater breeding densities would increase the likelihood of flycatcher visits to nest boxes occupied by tits.

## Experimental design

**Fig. 1:**
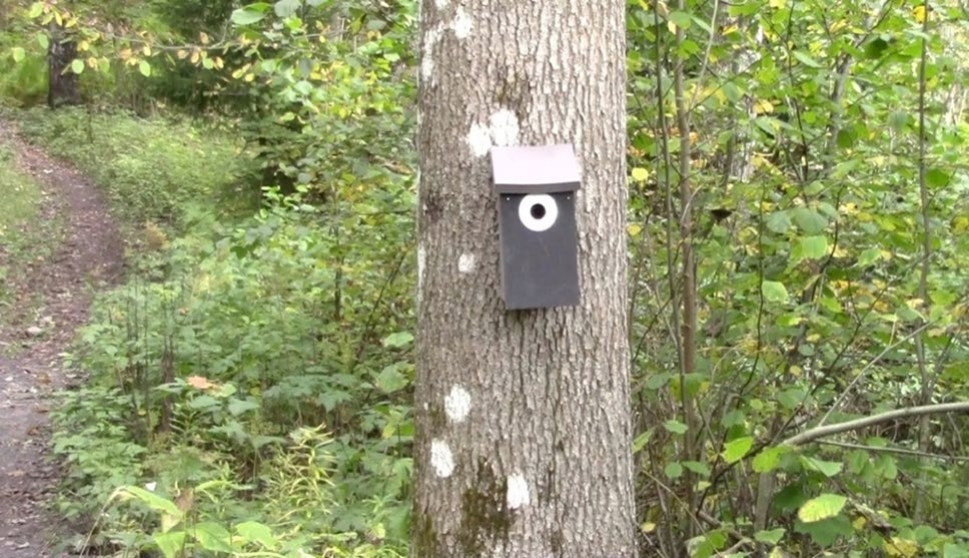
Nest box with a white circle painted around the opening hole, attached to a European Ash *Fraxinus excelsior* for illustration. The tree had several white, encrusting patches of lichen

Previous studies conducted by other researchers (see Forsman et al. 2018) have used an experimental design that involves nest boxes with a conspicuous white marking (termed a “symbol”, below) around the entrance. Following a similar protocol as Seppänen et al. (2011), when most Great Tits had finished nest-building and many had started egg-laying, we attached a thin, black-painted plywood faceplate to the front of the box on which we had painted a contrasting white circular symbol (75 mm diameter) around the entrance hole (Fig. 1), or a similar sized white triangle. Concomitantly, three empty nest boxes were erected; one box only 2–6 m from the tit box marked with a symbol opposite that of the tit box to simulate that the tit “demonstrator” had chosen a nest box with a particular symbol among the two available; and a dyad of boxes, one with a circle and the other with a triangle symbol, was placed ~ 25 m away and a few meters apart (termed the “25 m boxes”, below). The type of symbol attributed to tit nest boxes was randomized across trials. Here we define the “same symbol box” as the box placed at 25 m with a symbol matching that on the occupied tit box; the “different symbol box” was the 25 m box with a different symbol than on the occupied tit box. We recorded which of these two boxes the flycatchers chose to nest in. After a flycatcher settled, we recorded the date it began egg-laying and its clutch size. One female disappeared before any egg had been laid. We assigned a laying date to this nest from a comparison with females that had started nest-building at the same time. We caught 47 of the female flycatchers to age them as a yearling or older, following Svensson (1992).

We compared Great Tit clutch size between the trial sites where a Pied Flycatcher settled at one of the 25 m boxes (i.e. nest was found), versus locations where none settled. When a flycatcher settled, we compared its choice of nest box with the number of eggs and/or nestlings in the focal Great Tit nest on the day the flycatcher started nest-building. All Pied Flycatchers started egg-laying after the focal Great Tit had finished egg-laying (19.9 days ± 7.5, range 2–37, *n* = 54); only two started building when the tit was egg-laying, 33 when the tit was incubating, and 19 after tit hatching. It has been suggested that it is mainly the male flycatcher which assesses the tit clutch/brood and that the male has an important role in nest site choice (Forsman et al. 2018). Male Pied Flycatchers usually arrive several days before the females and hence can visit tit nests before hatching. Thus, we also compared the symbol choice of the Pied Flycatchers with the size of the completed clutch of the focal Great Tit.

### Video filming

We studied tit and flycatcher behaviour by video filming at the focal Great Tit nest box after the extra nest boxes had been provided and after the flycatchers had started to arrive. Results from filming at the 25 m boxes have been published elsewhere (Slagsvold and Wiebe 2021a). All tit nests filmed were first breeding attempts of the season. We used digital camcorders with 32 × optical zoom, on tripods placed about 6 m from the focal tit nest, ensuring that the box lid and the entrance hole were within the field of view so we could determine the sex of any Pied Flycatcher that approached the box and document whether it entered. We do not think the camera, which was hidden as much as possible behind trees and bushes, prevented the flycatchers from approaching the tit nest box; female and male Great Tits did not hesitate to enter their box during filming and Pied Flycatchers readily visited and entered both 25 m nest boxes which were filmed with the camera at a similar position (Slagsvold and Wiebe 2021a). As shown below, the Pied Flycatchers did not appear a long time after the start of a video. Similar positioning of video cameras has recently been used by others when studying flycatcher behaviour at nest boxes (Morinay et al. 2020a).

We could identify the sex of a tit by the width of its black breast stripe, and the sex of a flycatcher by the dorsal colour (Drost 1936). During the egg-laying period, we filmed at 21 Great Tit nests once, during 1–13 May in 2016 (10 nests), and during 15–31 May in 2017 (11 nests), between 6:45 and 17:00 h. Duration of filming was between 174 and 334 min (mean 222 ± 49). During the incubation period, we filmed at 53 tit nests once (13 each in 2016 and 2017, 27 in 2019). Duration of filming was between 112 and 375 min (210 ± 70). First egg laid for these tit nests was 18 April–30 May (5 May ± 10 days, *n* = 53), and they were filmed on 7 May–6 June (19 May ± 8 days, *n* = 53), which was 0–12 days after the focal tit had completed its clutch (6.8 days ± 2.9, *n* = 53). Preferably we should have filmed at the tit nests more than once to test for repeatability. However, we filmed at many nests, for a long time at each nest, and thus for a high number of hours in total (264 h). Very few Pied Flycatchers entered the nest boxes of the respective focal tits (see below) and thus even if we had filmed at a nest box more than once, the conclusion should hardly have changed.

The durations of periods inside and outside the nest box of the female tit during the incubation period were calculated using only periods when start and stop of the bouts were known. One trial was therefore excluded, leaving 52 females for analysis. We also recorded the rate of incubation feeding visits by male Great Tits (prey was delivered to the female at the opening or inside the box). For the Pied Flycatchers found nesting in the trial boxes, mean date of first egg was 27 May ± 6 (*n* = 54). Thus, the filming at the tit nests occurred when most flycatchers were prospecting for a nest site. To account for possible diurnal variation in flycatcher prospecting activity, we filmed the tit nests at variable times of day during the incubation period (06:56–15:52 h, *n* = 53) but mostly in the morning when Pied Flycatcher activity was assumed to be highest (mean start of filming at 8:35 h ± 86 min).

### Predictability of tit clutch size

Each year during 2016–19, we recorded laying date (assuming that one egg was laid per day) and clutch size of the Great Tits using our nest boxes in the study area at Dæli. The data for first clutches (*n* = 232) were analysed to study the variation, and thus the predictability, of Great Tit clutch size within and among years.

During egg-laying, Great Tits often cover their eggs with lining materials (Loukola et al. 2020a). Each year during 2016–19, when visiting tit nest boxes during egg-laying at Dæli, we removed the lid and first counted the eggs seen from above and then lifted away any covering materials to record the total number of eggs actually present. We used only first nest attempts and nests where final clutch size was known, and only one observation per nest, namely on the last visit to a focal nest during the laying period (closest to finishing laying), leaving a sample from 213 nests for the analysis. To test the method, we let two observers visit the same nest at the same time and independently note the number of eggs seen before the cover materials were removed. A close correlation was found between the percent of eggs seen by the two observers (Spearman rank correlation, *r*_*s*_ = 0.96, *n* = 15, *p* < 0.001).

### Data analysis

The Great Tits nesting at the “solitary” sites were not ringed but we assumed that the results from the dyad trials were independent across years because (1) the trials occurred at different sites in 2016 and 2017 followed by a year with no trials (2018). (2) For the period 2017–20, mean annual survival rate has been only about 40% for male and female Great Tits at Dæli (T. Slagsvold, unpublished data). (3) At the solitary sites, the nest boxes to attract Great Tits were erected in March and all trial boxes were taken down post-breeding in July. Thus, resident tits had to search for new cavities for roosting overwinter, possibly increasing the rate of divorce and breeding dispersal. Also, when the same locality was used in a different year, the four nest boxes of a trial were erected on new trees, each box at least 30 m from the respective previous sites. (4) Breeding site fidelity is low in female Pied Flycatchers (Lundberg and Alatalo 1992). In 2019, 14 of the 15 females that settled at a 25 m box were caught and ringed, but only one of them was recaptured among the 28 nesting females in a 25 m box in 2020. (5) Nest box choice of Collared Flycatchers *Ficedula albicollis*, a closely related species to the Pied Flycatcher, was unaffected by the symbols on the boxes in the previous year (Forsman et al. 2014).

With respect to the density of boxes used in our study, we tested whether flycatchers may have become confused by visiting more than one trial site with different symbols and tit clutch sizes. We noted the location, clutch size and the symbol painted on the focal tit nest box at trial sites where a Pied Flycatcher eventually nested in a 25 m box versus for the nearest Great Tit neighbour (with or without a Pied Flycatcher). The trials were divided in two groups based on the predictions from the SIIU hypothesis. If the symbol painted on the two tit boxes was the same and clutch size in both nests was below average for the season, or both were above average, the trial was termed “consistent”. This was also the case if both boxes had different symbols and different clutch sizes (one below and one above the average). The remaining trials were termed “confusing” (same symbols but different clutch sizes, or different symbols but similar clutch sizes).

We applied non-parametric tests (Spearman rank correlation, Chi-square, and Mann–Whitney *U* test) when variables were not normally distributed. Whether or not a flycatcher built a nest in a 25 m nest box with the same symbol as on the tit nest box was analysed by logistic regression (SPSS v. 25) treating tit clutch size and laying date of the focal flycatchers as a continuous variable, and study year and age of the female flycatcher as categorical variables. Statistical tests are two-tailed with an *α*-level of 0.05.

## Results

### Flycatcher choice of nest site

**Fig. 2:**
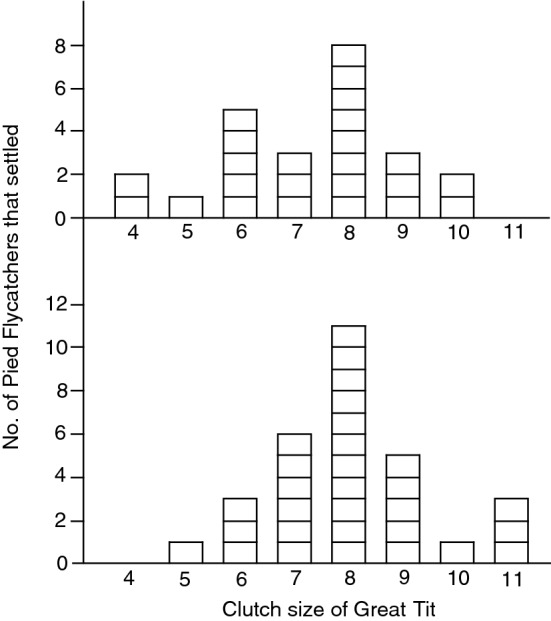
The frequency with which Pied Flycatchers chose nest boxes either bearing the same symbol as the "demonstrator" Great Tit nest box (top panel) or a different symbol (bottom panel). The number of choices is plotted in relation to clutch size of the tit. The prediction that the flycatchers would copy the symbol when the tit clutch size was large but not when it was small was not supported

In 2016, a pair of Pied Flycatchers settled in a 25 m box in 6 of 16 dyad trials, in 2017 in 3 of 13 trials, in 2019 in 15 of 28 trials, and in 2020 in 30 of 39 trials. The box type that female flycatchers chose for nest-building did not differ from random distribution with respect to the external symbol on the tit box; 24 built a nest in a same symbol box, 30 in a different symbol box (Chi-square test: χ^*2*^_1_ = 0.66, *p* = 0.42). Flycatchers did not choose the same symbol box when tit clutch size was relatively large (Fig. 2). A random choice of boxes still resulted if we only used trials at solitary sites (excluding data from 2016), or if we excluded the trials (*n* = 19) where the Pied Flycatcher started nest-building after the Great Tit eggs had hatched (Table 1).

**Table 1:** Content of the focal Great Tit nest when the Pied Flycatcher chose a nest box with the same, or a different, white marking as painted on the tit box

Study period	Number of eggs and/or nestlings in tit nest							
	Female age	Nesting stage^a^	Same symbol box_		Different symbol box		*t*-test	
			Mean ± SD	*n*	Mean ± SD	*n*	*t*	*p*
All		A	7.3 ± 1.7	24	8.0 ± 1.5	30	1.74	0.088
2017–2020	All	A	7.3 ± 1.7	21	8.1 ± 1.5	27	1.51	0.14
2016–2020	1st year	A	7.0 ± 1.8	9	7.7 ± 1.3	13	1.04	0.31
2016–2020	Older	A	8.0 ± 1.3	11	8.4 ± 1.6	14	0.73	0.48
2016–2020	All	A^b^	7.8 ± 1.1	13	8.0 ± 1.4	22	0.44	0.67
2016–2020	All	B	8.0 ± 1.4	24	8.3 ± 1.4	30	0.89	0.38
2016–2020	All	B^c^	8.1 ± 1.4	15	8.4 ± 1.7	14	0.54	0.54
2016–2020	All	B^d^	7.8 ± 1.4	9	8.2 ± 1.1	16	0.81	0.43

The trials are separated according to study period, age of the female flycatcher, and nesting stage

^a^A = The number of tit eggs and/or nestlings recorded on the day the female flycatcher started nest-building. B = The size of the completed tit clutch recorded during its incubation

^b^Only trials included where the flycatcher started nest-building before the tit eggs hatched

^c^Only trials where the respective symbols on the nest box and clutch sizes of the Great Tit and its nearest neighbour were consistent (see text)

^d^The remaining cases with symbols possibly causing confusion (see text)

A multiple logistic regression analysis of flycatcher symbol choice showed no significant effect of Great Tit nest content (χ^*2*^_1_ = 2.20, *n* = 47, *p* = 0.14), study year (χ^*2*^_3_ = 1.40, *p* = 0.70), age of female flycatcher (χ^*2*^_1_ = 0.75, *p* = 0.39), or date of first egg of the flycatcher (χ^*2*^_1_ = 2.44, *p* = 0.12). A model with only Great Tit nest content, age of female flycatcher, and their interaction showed no significant effects (Great Tit nest content: χ^*2*^_1_ = 1.60, *p* = 0.21; age of female flycatcher: χ^*2*^_1_ = 0.01, *p* = 0.90; interaction: χ^*2*^_1_ = 0.05, *p* = 0.82). Symbol choice was not affected by the distance to the nest of the nearest Great Tit neighbour (Great Tit nest content: χ^*2*^_1_ = 2.46, *p* = 0.12; distance: χ^*2*^_1_ = 0.17, *p* = 0.68). Likewise, models using only the complete clutch sizes for the focal Great Tits recorded during incubation found no difference in clutch size between trials where the flycatchers chose a box that did or did not have the same symbol as that painted on the tit box (Table 1, “nestling stage B”).

Flycatcher symbol choice was not affected by the clutch size of the nearest Great Tit, nor whether the symbol on this tit nest box was the same or different from the one on the focal tit nest box (focal Great Tit clutch size: χ^*2*^_1_ = 0.52, *p* = 0.47; neighbour tit clutch size: χ^*2*^_1_ = 0.25, *p* = 0. 61; symbol on neighbour: χ^*2*^_1_ = 0.53, *p* = 0. 47; interaction between neighbour tit clutch size and type of symbol on its box: χ^*2*^_1_ = 0.48, *p* = 0. 49). Whether nearest neighbour information was “consistent” or “confusing” did not affect the conclusion of no effect of tit clutch size on flycatcher symbol choice (Table 1). The clutch size of the Pied Flycatcher itself also did not differ according to whether it had copied the symbol (6.3 eggs ± 0.9, *n* = 23) or not (6.4 eggs ± 0.7, *n* = 28; *t* = 0.61, *p* = 0.55).

Pied Flycatchers did not seem to be attracted by pairs of Great Tits that had large clutches because final clutch size of the focal tit was similar when a pair of flycatchers settled (mean tit clutch size 8.2 eggs ± 1.4, *n* = 54) versus did not settle at a trial site (8.0 eggs ± 1.7, *n* = 39). A logistic regression analysis of flycatcher settlement showed no effect of tit nest content, study year, or their interaction (Great Tit nest content: χ^*2*^_1_ = 0.04, *p* = 0.85; year: χ^*2*^_3_ = 4.95, *p* = 0.17; interaction: χ^*2*^_3_ = 5.60, *p* = 0.13).

### Flycatcher visits to tit nests

During egg-laying, female Great Tits visited their own nest box in 19 of 21 trials and males visited it in six trials (Table 2). Of the flycatchers that appeared at the tit box, only three males approached or perched at the box opening and only one entered the box (for 9 s). During incubation, no female flycatcher appeared, and only 11 male flycatchers were seen; three males only hovered outside the box and/or perched briefly on the lid, whereas eight males perched at the opening of the tit box. In six of the eight cases, the female tit was inside the box and the flycatcher left within a few seconds.

**Table 2:** Number of trials, hours of filming, and observations of Great Tits and Pied Flycatchers during filmed trials at the occupied tit box. The trials were conducted during the egg-laying and incubation period of the tit

Variable	Egg-laying^a^	Incubation
Number of trials	21	53
Hours of filming	78	186
# Trials with male tit seen	6	44
# Trials with female tit seen	19	53
# Male flycatchers seen	3	11
# Male flycatchers entering nest box	1	2
# Female flycatchers seen	1	0
# Female flycatchers entering nest box	0	0

^a^From Slagsvold and Wiebe (2021a)

During incubation, the starting hour of a video did not differ between sites where a flycatcher appeared or did not appear (Mann–Whitney *U* test, *z* = -0.47, *n* = 53, *p* = 0.64) so flycatcher prospecting visits were not biased towards early or late hours of filming. However, the males that did appear, were first seen after on average 71 min (± 37, *n* = 11) of filming, on videos that lasted on average 226 min (± 49). Thus, the males appeared after on average 34% (± 21) of the filming period suggesting no fear of the cameras.

During incubation, only two of the flycatcher males entered the tit box (Table 2), occurring 30–31% into the filming period, and 8 and 41 min, respectively, after the female tit had left. Both males visited the box several times and soon started singing, apparently to attract a mate, but both departed the nest site when the tit returned. A comparison of the dorsal colouration based on Drost´s (1936) scale, and the size of the white forehead patch, between males seen at the 25 m boxes and at the nesting tit boxes suggested it was the same individual in six of eight cases. No Pied Flycatcher succeeded in taking over an active Great Tit nest and only a single dead male flycatcher was found in a Great Tit nest, apparently killed by the tit.

### Choice based in indirect cues

During incubation, the attentiveness of the female Great Tit at the nest was not correlated with her clutch size (*r*_s_ = -− 0.03, *n* = 53, *p* = 0.82), nor was rate of male incubation feeding correlated with it (*r*_s_ = -− 0.05, *n* = 53, *p* = 0.73). In the study area at Dæli, there was strong variation in annual mean clutch size of Great Tits, ranging from 7.6 to 9.2 eggs. Clutch size (range 3–12 eggs) was not associated with date of first egg laid during the four years of the study (ANCOVA, year effect, *F*_*3,227*_ = 9.16, *p* < 0.001; laying date, *F*_*1,227*_ = 0.64, *p* = 0.43).

### Great Tit clutch size and egg covering

**Fig. 3:**
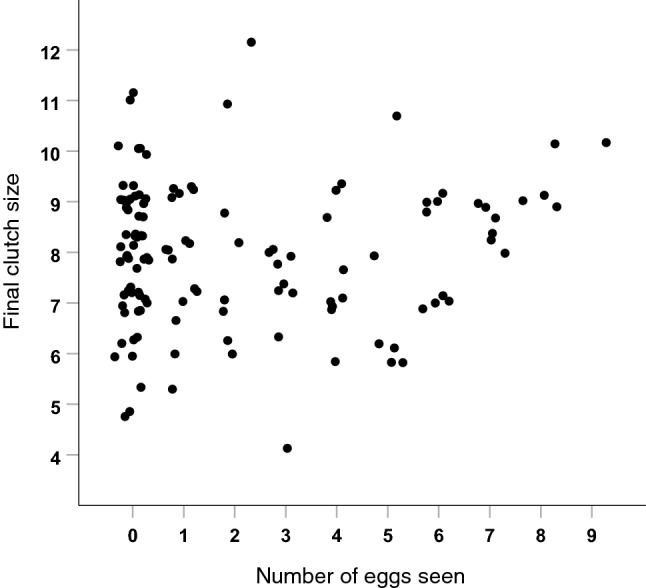
There was no correlation between the final clutch size of Great Tits in relation to the number of eggs seen by a human observer when visiting a nest during the egg-laying period. Only visits when there were 1–3 eggs yet to be laid per clutch are included (*n* = 123)

The Great Tit nests we visited during egg-laying at Dæli (*n* = 213) had eventual full clutches of 8.2 ± 1.4 eggs but the range was great (4–12). Thus, it would only be possible to conclude that a clutch was above average if there were at least nine eggs visible during a nest check. In only 3% of our haphazard visits to tit nests during the egg-laying period were there ≥ 9 eggs and the number of these actually visible was further reduced because of egg covering. On average, 58% of the clutch had been laid when we checked nests during the egg-laying period for the last time but only 34% ± 41 of eggs present were visible. During these visits, all eggs were seen in only 22% of the nests and in 50% of nests no eggs were visible at all. There was no correlation between final clutch size and the percent of eggs laid that were seen during a visit (Spearman rank correlation, *r*_*s*_ = -− 0.07, *n* = 213, *p* = 0.34). Final clutch size could not even be predicted if visiting a tit nest 1–3 days before the final egg was laid (jitter plot in Fig. 3; *r*_*s*_ = 0.03, *n* = 123, *p* = 0.70), nor even on the day the last egg was laid (*r*_*s*_ = 0.26, *n* = 31, *p* = 0.15).

### Incubation rhythms of tits

**Fig. 4:**
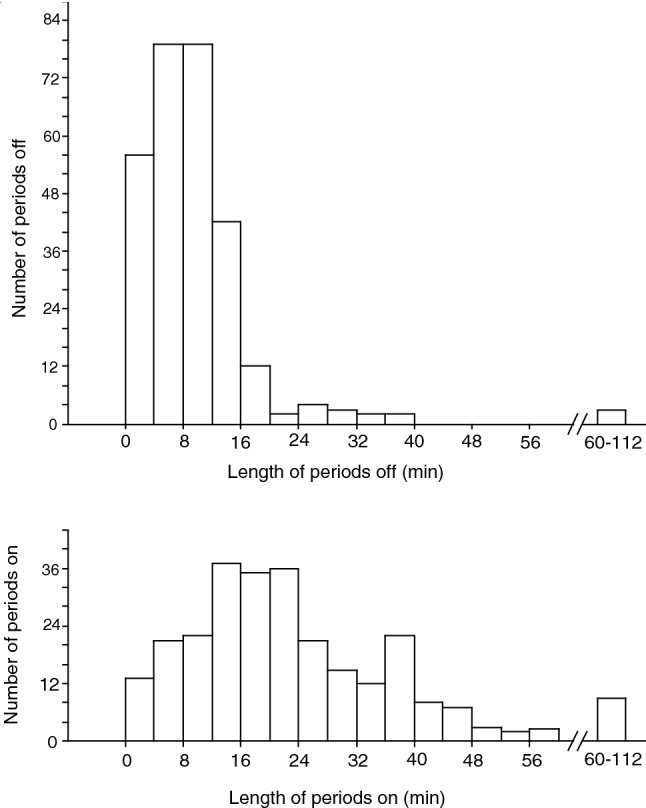
Frequency of the duration of periods spent outside (top panel, *n* = 284) and inside (bottom panel, *n* = 266) the nest box by incubating female Great Tits. Data from 52 nests filmed for a total of 184 h

Median length of incubation bouts per female ranged from 0.3 to 112 min (*n* = 51), whereas bouts outside the box ranged from 2 to 49 min (*n* = 52). For all incubation bouts combined, the median was 21 min (Fig. 4 top panel; *n* = 266 bouts) whereas bouts outside the box were short and also of unpredictable duration (Fig. 4 bottom panel; median 8 min, *n* = 284). The female tit spent on average 68% ± 17 (*n* = 53) of the time inside her nest box. This attentiveness declined with hour of the day (onset of filming; Spearman rank correlation, *r*_*s*_ = -0.39, *n* = 53, *p* = 0.005). Most male Great Tits visited their box, apparently with food for the female, at a median rate of 2.1 visits/h (range 0–9.3, *n* = 53) when the female was inside.

## Discussion

### No evidence that flycatchers used social information

Our test of the SIIU hypothesis failed to support the prediction that Pied Flycatchers would copy the external appearance of the nest cavity of a demonstrator tit when its clutch or brood size is larger than average but not when it is smaller, a pattern reported in two other countries (Finland and Latvia) when using a similar experimental design (Seppänen et al. 2011; Loukola et al. 2013). In our study in Norway, we found only random settlement by flycatchers with respect to the symbol on the tit box and tit clutch size (Table 1). In 2016 and 2017, we also video filmed at the 25 m boxes. However, the male flycatchers that settled did not display more at the same symbol box than at the different symbol box (Slagsvold and Wiebe 2021a). Others have reported that the nest box choice of pied and collared flycatchers is affected by markings added on nest boxes occupied by tits but those studies (e.g., Forsman and Seppänen 2011; Morinay et al. 2020a) used a different design than Seppänen et al. (2011) and so the results are not directly comparable. None of these studies have shown that prospecting flycatchers actually entered tit nest cavities to assess the contents directly.

According to the SIIU, the nest box choice of the Pied Flycatcher should be related to the number of eggs/nestlings in the focal tit nest on the day the female flycatcher starts nest-building, which we showed in Table 1. However, Great Tits often lay a large clutch and once nestlings hatch, they lie on top of each other in a pile in the nest cup. Thus, it seems improbable that a prospecting flycatcher could get an accurate assessment of the fecundity of the tit based on looking at nestlings from the box entrance and even if entering. However, it is also unlikely that flycatchers can assess final tit clutch size during the egg-laying period because many of these eggs are covered with lining materials as shown in the current study, and such egg-covering behaviour has been reported from all parts of the Great Tits’ breeding range (Loukola et al. 2020a). Regardless of whether there were only eggs or nestlings, however, we found no evidence that flycatchers of either sex relied on such information.

In our study, Pied Flycatchers did not prefer to settle near Great Tit nests with large rather than small clutches, nor did they take an external characteristic of the tit’s nest cavity into account when choosing a nest site. The discrepancy between our results and other studies is difficult to explain because our data were collected over a 4-year period and should be representative. We have no a priori reason to expect that the SIIU hypothesis is population or habitat-specific because flycatchers must be able to assess tit clutch and brood size to use it as a cue. Our study area was at a similar latitude as those in Finland and Latvia, and thus the time constraint on onset of breeding by flycatchers should be similar. Possibly prospecting flycatchers use indirect cues to assess tit quality, but we do not see why such cues would differ in importance among populations. Great Tits may defend more than one cavity on their territory for renesting and we have suggested a mechanism by which such tit aggression may result in a non-random choice of boxes with different painted symbols (Slagsvold and Wiebe 2017). An experimental presentation of a Pied Flycatcher "intruder" at the 25 m boxes elicited defence of the boxes by the focal Great Tits as predicted, but the aggression did not differ between the same and the different symbol boxes (Slagsvold and Wiebe 2021a) and we cannot envision why the pattern of aggression would differ between study areas.

Although the distances between our trial sites were shorter on average than those in Finland and Latvia (Seppänen et al. 2011) this does not seem to have caused different outcomes in the studies. First, both in Finland (Forsman et al. 2018) and in Norway (present study), Pied Flycatchers rarely entered tit nests after egg-laying had finished and thus they seem unable to assess clutch size in any Great Tit nest at high or low densities. Second, separating our data in two groups of “consistent” and “confusing” cases, did not affect the results. Finally, previous studies have shown that when male and female Pied Flycatchers have found a suitable nest site, they only continue to search within a restricted area due to time constraints and intraspecific competition (Slagsvold 1986; Dale and Slagsvold 1996).

### Why do not flycatchers usually enter tit nest boxes?

Flycatchers rarely entered tit nests after the completion of egg-laying in our study and in one in Finland (Forsman et al. 2018). This reluctance to enter makes sense because Great Tits are known to kill pied and collared flycatchers that enter their nest boxes in all areas of sympatry (Slagsvold 1975; Merilä and Wiggins 1995; Ahola et al. 2007; Forsman et al. 2018; Samplonius and Both 2019). Flycatchers can only assess the contents of tit nests when the owner is absent but females were inside the box in 6 of 8 cases that a male Pied Flycatcher perched at the entrance hole. This was close to random expectation because an incubating female tit spent on average 68% of the time inside her nest box. Thus, male flycatchers did not seem to wait and watch the tit box from a distance to time any next box entrances according to the departure of the female tit.

We did not film at sunrise or in the evenings, but there was no morning bias to the observed visits of Pied Flycatchers and in any case, female tits are very attentive to clutches early and late in the day when the ambient temperature is cool and the eggs chill rapidly (Haftorn 1988). Our filming showed that the female tit's off-bouts during incubation were short and unpredictable (Fig. 3, and see also Haftorn 1981; Álvarez and Barba 2014; for similar results). Male Great Tits often remained near the nest box (as evidenced by males heard singing nearby on the videos) and this would also make it difficult for any intruder to visit the cavity unnoticed. In Finland, several flycatchers were found dead in tit nests, but none during the incubation period (Forsman et al. 2018) which is also indicative of infrequent entries by flycatchers after clutch completion.

Pied Flycatchers may enter tit nests during the egg-laying period (Forsman et al. 2018) but we suggest that the most parsimonious explanation is that the flycatchers are searching for a nest site of their own but they may be killed occasionally when prospecting, and in particular when available cavities are few (Slagsvold 1978; Merilä and Wiggins 1995). Indeed, the two male flycatchers that we saw entering a tit nest box during the incubation period started to sing beside the box and visited it several times although it presumably would take only one entry to assess the number of uncovered tit eggs. The presence of existing nest material in boxes may be attractive to flycatchers which, to save time and energy, readily build their own nests over abandoned or old tit nests (Orell et al. 1993; Loukola et al. 2014). Furthermore, the strong annual variation in Great Tit clutch size (up to 1.6 eggs on average between two breeding seasons) means that several tit nests would need to be visited within a breeding season for a prospecting flycatcher to be able to assess whether a tit clutch is relatively small or large but data show that male and female Pied Flycatchers visit few nest cavities before settling (Slagsvold 1986; Dale and Slagsvold 1996). The SIIU hypothesis predicts that it may be difficult for prospecting flycatchers to use tit clutch size as a cue in years when flycatchers arrive and settle early after migration when many tits may not yet have initiated or completed their clutches (Morinay et al. 2018; Szymkowiak 2019). However, in our study, the peak period of flycatcher arrival and settlement occurred when we filmed the tit nests, so a lack of active tit nests cannot explain the scarcity of entrances by flycatchers.

### Indirect cues of clutch size and avenues of further investigation

We suggest that the constraints on flycatchers entering tit nest boxes to assess clutch size will apply widely and would make the use of indirect cues safer than the use of direct cues. Our results showed, however, that flycatchers did not use the laying date of the tit or incubation behaviour (female attentiveness, male incubation feeding) as indirect cues of its final clutch size. The age and body mass of tits were also not associated with choice by flycatchers in the previous study by Seppänen et al. (2011). Discrepancies between results arising from different study areas strongly suggests that further replication of symbol trials should be done to determine whether random nest site choice by flycatchers in relation tit clutch size is more common across their range. In populations where non-random nest selection has been reported, further studies of indirect cues of tit quality, such as their song (Morinay et al. 2020b), timing of settlement and egg-laying (Samplonius and Both 2017), aggression and availability of alternative nest sites may be warranted (Slagsvold and Wiebe 2021a).

Beyond the proximate challenge of assessing clutch size, an ultimate problem with the present version of the SIIU hypothesis is to understand what a prospecting flycatcher gains from using information about the external traits of a Great Tit nest site. Forsman et al. (2018, p. 2) called the white markings painted around the nest box hole “unnatural symbols that have no ecological relevance” but we noted that the markings resemble patterns frequently found on the bark of tree trunks in the wild (Fig. 1). However, the lichen patches are highly variable in size and shape as are the interior dimensions of natural cavities used by birds (Wiebe 2001; Maziarz et al. 2015). An untested assumption of the SIIU is that prospecting birds can distinguish and remember the size and shapes of the external markings on a number of nest cavities. Results from a recent study on blue tits *Cyanistes caeruleus* on our study area cast doubt on this assumption because females sometimes built nests and laid eggs in adjacent boxes painted with different symbols as if they could not locate the correct entrance (Slagsvold and Wiebe 2021b).

Experiments show that nest site choice of Pied Flycatchers is strongly dependent on interior cavity quality, including moisture and presence of nest materials and ectoparasites (Slagsvold and Lifjeld 1988; Loukola et al. 2014; Breistøl et al. 2015). We can conceive of no adaptive reason why a cavity nester would be motivated to copy the external appearance of another species’ nest site. More likely, nest site quality is assessed by direct inspection of available, undefended sites, where a quick visit may be sufficient (Dale and Slagsvold 1996), in contrast to assessing, for instance, habitat quality (presence of food and predators, nest and roosting sites) where heterospecific social learning may be important (Danchin et al. 2004; Parejo et al. 2005; Szymkowiak et al. 2017). Solitary bees may take into account an external symbol on nest site of another bee species when choosing their own nest site but the symbol in that case could be associated with a fitness benefit, namely the risk of being parasitized (Loukola et al. 2020b).

Our findings call into question the idea that assessing directly the clutch and brood size of heterospecifics is a general mechanism that birds use to obtain information about the quality of nest sites. The danger of inspecting nest sites of heterospecifics may be common across an array of species. Furthermore, the external appearance of the nest of a heterospecific species does not seem to be an attribute that confers reproductive benefits so it is unclear why it is a feature that should be copied. Therefore, similar experiments of nest site choice should be replicated more widely across the geographic range of Great Tits and Pied Flycatchers and should also include other species.

## Data Availability

The datasets are available from the corresponding author on reasonable request.
